# Supplementary material for: Fishermen Interviews: A Cost-Effective Tool for Evaluating the Impact of Fisheries on Vulnerable Sea Turtles in Tunisia and Identifying Levers of Mitigation
Source: Animals (Basel). 2023 May 4;13(9):1535. doi: 10.3390/ani13091535 (PMC10177481; doi:10.3390/ani13091535)
Supplement: Supplementary file 1 [file animals-13-01535-s001.zip › animals-2311095-supplementary.pdf]

Supplementary Material

Table S1. Full—Zero-inflated negative binomial GLMM results—fishing nets: factors included in the model and their significance

	**N_loggerhead_month**			
Predictors	*Incidence Rate Ratios*	*CI*	*p*	*df*
**Count Model**				
(Intercept)	0.04	0.00–0.55	**0.016**	Inf
Gulf [Hammamet]	0.54	0.29–1.01	0.055	Inf
Type of nets [Encircling nets]	1.20	0.28–5.18	0.805	Inf
Type of nets [Bony fish gillnets]	0.58	0.31–1.08	0.089	Inf
Type of nets [Trammel nets]	0.39	0.22–0.70	**0.002**	Inf
Type of nets [Garrasya]	10.01	3.00–33.43	**<0.001**	Inf
Type of nets [Gattatya]	2.86	0.91–8.95	0.072	Inf
Ral inf Distance entre deux plombs, cm	1.00	1.00–1.01	0.408	Inf
Effort operation month Length number of pieces [log]	1.41	1.09–1.84	**0.010**	Inf
Gulf [Gabes] * cos(PseudoFourier)	0.64	0.49–0.85	**0.002**	Inf
Gulf [Hammamet] * cos(PseudoFourier)	0.65	0.32–1.31	0.226	Inf
Gulf [Gabes] * sin(PseudoFourier)	1.18	0.96–1.45	0.123	Inf
Gulf [Hammamet] * sin(PseudoFourier)	1.04	0.64–1.71	0.861	Inf
(Intercept)	6.63	3.75–14.97		
**Zero-Inflated Model**				
(Intercept)	8.79	0.33–233.55	0.194	Inf
Gulf [Hammamet]	3.81	1.85–7.84	**<0.001**	Inf
Type of nets [Encircling nets]	0.12	0.00–4.26	0.247	Inf
Type of nets [Bony fish nets]	9.79	4.36–21.97	**<0.001**	Inf

Type of nets [Trammel nets]	3.82	1.74–8.36	**0.001**	Inf
Type of nets [Garrasya]	0.92	0.14–5.87	0.926	Inf
Type of nets [Gattatya]	2.36	0.63–8.87	0.203	Inf
Foot rope distance between two weights, cm	0.99	0.99–1.00	**0.041**	Inf
Effort operation month *Length* number of pieces [log]	0.81	0.58–1.12	0.199	Inf
Gulf [Gabes] * cos(PseudoFourier)	2.39	1.59–3.61	**<0.001**	Inf
Gulf [Hammamet] * cos(PseudoFourier)	5.12	2.08–12.58	**<0.001**	Inf
Gulf [Gabes] * sin(PseudoFourier)	0.73	0.53–1.00	**0.047**	Inf
Gulf [Hammamet] * sin(PseudoFourier)	1.09	0.57–2.06	0.800	Inf
**Random Effects**				
σ^2	2.17			
$\tau_{00\ Port:Boat.ID}$	1.11			
ICC	0.34			
N_{Port}	19			
$N_{Boat.ID}$	372			
Observations	3795			
Marginal R^2/Conditional R^2	0.120/0.419			

Table S2. GLMM for fishing nets: model selection based on AIC (response variables: number of loggerheads/month; zero-inflated negative binomial; random factors: Port:ID.Vessel; fishing effort as offset: N_operation_month *Net_Length*NumberofNetPieces)

Covariables included in the model	AIC	ΔAIC	Ak[illegible] we[illegible]
Gulf + Fishing_Net_Type + Distance Between Two Sinkers (Lower Sling)	4926.867	185,798	[illegible]
Gulf + Gulf:cos(PseudoFourier) + Gulf:sin(PseudoFourier) + Distance Between Two Sinkers (Lower Sling)	4847.036	105,967	[illegible]
Gulf:cos(PseudoFourier) + Gulf:sin(PseudoFourier) + Fishing Net Type + Distance Between Two Floats (Upper Sling)	4777.421	36,352	[illegible]
Gulf + Gulf:cos(PseudoFourier) + Gulf:sin(PseudoFourier) + Fishing Net Type + Depth + Distance Between Two Floats (Upper Sling)	4751.291	10,222	[illegible]
Gulf + Gulf:cos(PseudoFourier) + Gulf:sin(PseudoFourier) + Fishing Net Type + Distance Between Two Floats (Upper Sling)	4749.340	8,271	[illegible]

Gulf + Gulf:cos(PseudoFourier) + Gulf:sin(PseudoFourier) + Fishing Net Type + Depth + Distance Between Two Floats (Upper Sling) + Distance Between Two Sinkers (Lower Sling)	4744.089	3,02	[illegible]
Gulf + Gulf:cos(PseudoFourier) + Gulf:sin(PseudoFourier) + Fishing Net Type + Depth + Distance Between Two Sinkers (Lower Sling)	4743.056	1,987	[illegible]
Gulf + Gulf:cos(PseudoFourier) + Gulf:sin(PseudoFourier) + Fishing Net Type + Distance Between Two Floats (Upper Sling) + Distance Between Two Sinkers (Lower Sling)	4742.128	1,059	[illegible]
Gulf + Gulf:cos(PseudoFourier) + Gulf:sin(PseudoFourier) + Fishing Net Type + Distance Between Two Sinkers (Lower Sling)	4741.069	0	[illegible]

Table S3. Full—zero-inflated negative binomial GLMM results—longlines: factors included in the model and their significance

	**Dependent variable**		
Predictors	*Incidence Rate Ratios*	*CI*	*p*
**Count Model**			
(Intercept)	0.00	0.00–0.00	**<0.001**
Gulf [Hammamet]	0.28	0.10–0.82	**0.020**
Longlines type [Pelagic]	2.84	1.44–5.59	**0.003**
Number of weights km	0.91	0.78–1.05	0.204
Hook size [1st degree]	0.00	0.00–0.01	**0.008**
Hook size [2nd degree]	0.00	0.00–0.78	**0.044**
Gulf [Gabes] * cos(PseudoFourier)	0.94	0.46–1.90	0.862
Gulf [Hammamet] * cos(PseudoFourier)	0.39	0.27–0.57	**<0.001**
Gulf [Gabes] * sin(PseudoFourier)	1.37	0.71–2.66	0.352
Gulf [Hammamet] * sin(PseudoFourier)	0.56	0.40–0.78	**0.001**
(Intercept)	232.50	14.50–66279.10	
**Zero-Inflated Model**			
(Intercept)	1.20	0.66–2.21	0.549
Longlines type [Pelagic]	0.23	0.10–0.51	**<0.001**

**Random Effects**	
σ^2	2.53
τ_{00} Port:Boat.ID	2.16
ICC	0.46
N Port	11
N Boat.ID	90
Observations	429
Marginal R^2/Conditional R^2	0.798/0.891

Table S4. GLMM for longlines: model selection based on AIC
(zero-inflated negative binomial; response variables: number of loggerheads/month; random factors: Port:ID.Vessel; fishing effort as offset: N_operation_month * number of hooks)

Covariables included in the model	AIC	ΔAIC	Akaike weight
Gulf + Gulf:cos(PseudoFourier) + Gulf:sin(PseudoFourier)	1258.176	63.890	0
Gulf + Gulf:cos(PseudoFourier) + Gulf:sin(PseudoFourier) + Longline Type	1212.391	18.105	0
Gulf + Gulf:cos(PseudoFourier) + Gulf:sin(PseudoFourier) + Longline Type + Longline Type: Number Of Floats/km	1209.485	15.199	0
Gulf + Gulf:cos(PseudoFourier) + Gulf:sin(PseudoFourier) + Longline Type + Longline Type: Number Of Floats/km + Number Of Sinkers/km + Longline Type: Branchline Length	1204.105	9.819	0.005
Gulf + Gulf:cos(PseudoFourier) + Gulf:sin(PseudoFourier) + Longline Type + Number Of Sinkers/km + poly(Hook Size, 2) + Branchline Diameter + Buoys Type + Bait + Branchline Diameter	1203.786	9.500	0.006
Gulf + Gulf:cos(PseudoFourier) + Gulf:sin(PseudoFourier) + Longline Type + Longline Type: Number Of Floats/km + Number Of Sinkers/km + Number of Buoys/km	1203.100	8.814	0.009
Gulf + Gulf:cos(PseudoFourier) + Gulf:sin(PseudoFourier) + Longline Type + Longline Type: Number Of Floats/km + Number Of Sinkers/km	1201.142	6.856	0.023
Gulf + Gulf:cos(PseudoFourier) + Gulf:sin(PseudoFourier) + Longline Type + Number Of Sinkers/km	1198.054	3.768	0.110
Gulf + Gulf:cos(PseudoFourier) + Gulf:sin(PseudoFourier) + Longline Type + Longline Type: Number Of Floats/km + Number Of Sinkers/km + poly(Hook Size, 2)	1197.601	3.315	0.136
Gulf + Gulf:cos(PseudoFourier) + Gulf:sin(PseudoFourier) + Longline Type + Number Of Sinkers/km + poly(Hook Size, 2)	1194.286	0	0.712

Table S5. Full—zero-inflated (hurdle) negative binomial GLMM results—trawls: factors included in the model and their significance

	**Dependent variable**		
Predictors	*Incidence Rate Ratios*	*CI*	*p*
**Count Model**			
(Intercept)	0.01	0.00–0.09	**<0.001**

Gulf [Hammamet]	0.22	0.11–0.45	**<0.001**
Arm length [1st degree]	112.04	0.28–44993.89	0.123
Arm length [2nd degree]	0.03	0.00–6.39	0.198
Length of footrope	1.10	1.02–1.19	**0.014**
Length of headrope	0.90	0.83–0.98	**0.017**
Mesh size of cod end	1.05	0.97–1.15	0.245
Haul duration (duration)	0.79	0.54–1.16	0.228
Gulf [Gabes] * cos(PseudoFourier)	0.54	0.46–0.63	**<0.001**
Gulf [Hammamet] * cos(PseudoFourier)	0.34	0.18–0.62	**<0.001**
Gulf [Gabes] * sin(PseudoFourier)	1.29	1.05–1.58	**0.014**
Gulf [Hammamet] * sin(PseudoFourier)	1.13	0.56–2.29	0.727
(Intercept)	8.43	4.46–20.88	
**Zero-Inflated Model**			
(Intercept)	3.00	0.81–11.08	0.099
Boat ID [1096 HS]	0.00	0.00–Inf	0.999

Random Effects

σ^2	0.58
$\tau_{00\ Boat.ID}$	0.51
ICC	0.47

$N_{Boat.ID}$	63
Observations	637
Marginal R^2/Conditional R^2	0.454/0.709

Table S6. GLMM for trawls, model selection based on AIC
(zero-inflated hurdle-type negative binomial; response variable: number of loggerheads/month; random factor: ID.Vessel; fishing effort as offset: N_operation_month * haul duration)

Covariables included in the model	**AIC**	**ΔAIC**	**Akaike weight**
Gulf + Gulf:cos(PseudoFourier) + Gulf:sin(PseudoFourier) + poly(Arm Length, 2) + Head Rope Length + Cul-de-sac Mesh Size + poly(Otter Board Length, 2)	2137.823	6.422	0.01871900
Gulf + Gulf:cos(PseudoFourier) + Gulf:sin(PseudoFourier) + poly(Arm Length, 2) + Foot Rope Length + Cul-de-sac mesh size + poly(Otter Board Length, 2)	2137.478	6.077	0.02224326
Gulf + Gulf:cos(PseudoFourier) + Gulf:sin(PseudoFourier) + poly(Arm Length, 2) + Foot Rope Length + Head Rope Length + Cul-de-sac Mesh Size + poly(Otter Board Length, 2)	2133.851	2.450	0.13639283
Gulf + Gulf:cos(PseudoFourier) + Gulf:sin(PseudoFourier) + Foot Rope Length + Head Rope Length + Cul-de-sac Mesh Size + poly(Otter Board Length, 2)	2133.429	2.028	0.16843319
Gulf + Gulf:cos(PseudoFourier) + Gulf:sin(PseudoFourier) + poly(Arm Length, 2) + Foot Rope Length + Head Rope Length + poly(Otter Board Length, 2)	2133.189	1.788	0.18990789
Gulf + Gulf:cos(PseudoFourier) + Gulf:sin(PseudoFourier) + poly(Arm Length, 2) + Foot Rope Length + Head Rope Length + Cul-de-sac Mesh Size	2131.401	0.000	0.46430383
